# Factors Associated with Substance Use and Physical Activity Among German University Students 20 Months into the COVID-19 Pandemic

**DOI:** 10.1007/s10935-025-00865-8

**Published:** 2025-07-05

**Authors:** S. M. Helmer, C. Buck, P. M. Matos Fialho, C. R. Pischke, C. Stock, E. Heumann, H. Zeeb, S. Negash, R. T. Mikolajczyk, Y. Niephaus, H. Busse

**Affiliations:** 1 Working Group Evidence-Based Public Health, Institute of Public Health and Nursing Research, University of Bremen, Bremen, Germany; 2 Leibniz Institute for Prevention Research and Epidemiology - BIPS, Bremen, Germany; 3 Institute of Medical Sociology, Centre for Health and Society, Medical Faculty, Heinrich-Heine-University Duesseldorf, Duesseldorf, Germany; 4 Institute of Health and Nursing Science, Charité – Universitätsmedizin, Corporate Member of Freie Universität and Humboldt Universität zu Berlin, Berlin, Germany; 5 Unit for Health Promotion Research, University of Southern Denmark, Esbjerg, Denmark; 6 Health Sciences Bremen, University of Bremen, Bremen, Germany; 7 Institute for Medical Epidemiology, Biometrics and Informatics, Interdisciplinary Center for Health Sciences, Medical School of the Martin Luther University Halle-Wittenberg, Halle (Saale), Germany; 8 Partner site Halle-Jena-Magdeburg, German Center for Mental Health (DZPG), Halle, Germany; 9 Department of Social Sciences, University of Siegen, Siegen, Germany

**Keywords:** Health risk behaviour, University students, COVID-19, Substance use, Physical activity

## Abstract

Substance use (SU) and physical activity (PA) among university students changed with the beginning of the COVID-19 pandemic. In this study, we examined factors associated with SU and PA and profiles of these health behaviours in university students in Germany 20 months into the pandemic. A cross-sectional study on student well-being during the COVID 19-pandemic was conducted in October/November 2021 at five German universities. 7203 students completed the web-based survey. Smoking, binge drinking, cannabis use, and moderate and vigorous PA served as outcomes. Twenty months into the pandemic, 17% of students reported smoking, 19% binge drinking and 6% cannabis use at least once during the last week. 13% reported low moderate and 35% low vigorous PA in the last week. Students perceiving studies to be more important than other activities showed lower odds for SU. Depressive symptoms were associated with a higher chance of smoking (OR 1.07; 95%-CI 1.06–1.09 per one unit on the CESD-8-scale) and cannabis use (1.07; 95%-CI 1.04–1.09), low moderate (1.09; 95%-CI 1.07–1.11) and low vigorous (1.06; 95%-CI 1.05–1.07) PA. The ‘health protective behaviour’-profile with high overall PA and low SU was the most common one found. Among other factors, perceiving studying to be less important than other activities and reporting depressive symptoms appear to play a role in reporting risky health behaviours. Prevention programmes should therefore be integrated in the university setting and address mental health issues and health behaviours simultaneously.

**Supplementary Information:**

The online version contains supplementary material available at 10.1007/s10935-025-00865-8.

## Introduction

University students are in a vulnerable life stage regarding health risk behaviours such as Substance Use (SU) and low Physical Activity (PA). Data assessed prior to the COVID-19 pandemic revealed particularly high-risk behaviours in this population, such as sedentary behaviour (Castro et al., 2020) and engagement in alcohol use (Davoren et al., 2016). During the COVID-19 pandemic, strict measures, such as physical distancing rules and closures of restaurants, cafeterias and libraries were implemented. These measures, intended to reduce the spread of the virus, were associated with stressors for many people, such as frustration or boredom due to reduced contact with others and loss of (social) routines (Brooks et al., 2020).

European research conducted at the beginning of the pandemic suggested that the actions to prevent the spread of COVID-19 had an impact on the SU and PA of university students, but findings were inconsistent. Some studies among European college and university students showed a decrease in students self-reported binge drinking during the early pandemic compared prior to the pandemic (Heradstveit et al., 2022; Rubio et al., 2023; Tavolacci et al., 2021; Tholen et al., 2024), whereas others found no difference in binge drinking between these time points (Zysset et al., 2022). Inconsistencies were also reported with regard to smoking during the early pandemic compared to prior to COVID-19 pandemic: whilst some reported a decrease (Larsson et al., 2022; Tholen et al., 2024), other studies revealed no change in smoking behaviour (Kale et al., 2022; van Hooijdonk et al., 2022) but increasing rates of quit attempts (Kale et al., 2022) or even increasing tobacco use rates (Vanderbruggen et al., 2020). With regard to cannabis use, conflicting findings also were found: a multinational sample displayed decreasing trends after the pandemic (Tavolacci et al., 2021; Tholen et al., 2022) while no significant changes were reported in a university student sample from Belgium (Vanderbruggen et al., 2020).

In university students in Germany, binge drinking was found to be highly prevalent prior to the COVID-19 pandemic among students, but many students reduced their binge drinking behaviour during the pandemic (Busse et al., 2021). Smoking and cannabis use remained almost unchanged from pre-pandemic levels (Busse et al., 2021). 

With regard to changes in PA in university students, studies reported mixed results during the COVID-19 pandemic. Evidence from systematic reviews conducted during the beginning of the pandemic demonstrated that PA levels have decreased among university students in general (López-Valenciano et al., 2020; Rivera et al., 2021), however, some studies also found increasing rates in PA during this time (Goncalves et al., 2021; Romero-Blanco et al., 2020). Also 9 months after the beginning of the pandemic reduction in PA levels were found (Savage et al., 2021). A more detailed look at the changes showed that while total physical activity had decreased, certain sports such as high-intensity interval training or mind–body activities have been practiced more frequently (Rodríguez-Larrad et al., 2021).

A survey among students in Germany conducted early during the pandemic indicated that changes in PA were inconsistent, with some students showed higher and some students lower PA levels compared to prior to the pandemic (Busse et al., 2021). Another German study revealed that the majority of students self-reported a worsened fitness level in the early pandemic (Gewalt et al., 2022).

There is currently a lack of data on SU and PA among students at a later stage of the pandemic. A Spanish cohort study has shown a decline in prevalence of binge drinking and cannabis use in the early pandemic period, followed by an increase at a later stage of the pandemic (Botella-Juan et al., 2022, 2024). A Swedish cohort study showed a slight decline in smoking behaviour in the early pandemic, which persists in the late pandemic (Larsson et al., 2022). For students in Germany, multiple cross-sectional studies on alcohol consumption are available, which also depicted similar patterns (Dogan-Sander et al., 2021). In terms of PA, a Swedish cohort study showed that exercise declined at the beginning of the pandemic and that even greater differences in the late pandemic existed, but conversely increases in daily activities were found that persisted into the late pandemic (Larsson et al., 2022). A better understanding of SU and PA in university students in Germany after a longer time period under these specific circumstances seems required as this information can help prevention scientists to monitor specific health behaviours, to identify high-risk groups in crises situations and to develop interventions accordingly.

Therefore, the purpose of this study was to contribute to the understanding of factors associated with involvement in SU and/ or PA among university students in Germany in a later stage (the fourth wave) of the pandemic. Moreover, we aimed to present profiles of behaviour patterns in this group to inform future prevention programmes. The research questions guiding this article relate to a later stage of the pandemic (20 months into the pandemic) and were formulated as:

Research question 1 (RQ1): To what extent do university students in Germany report engagement in licit and illicit SU as well as low moderate and vigorous PA?

RQ 2: Are socio-demographic factors, study-related factors and/or depressive symptoms, having a trusted person and boredom associated with engagement in SU and/or PA in university students in Germany?

RQ 3: Which profiles of the considered health behaviours can be identified among university students in Germany? 

## Methods

### Data

The analysis is based on data from the COVID-19 German Student Well-being Study (C19 GSWS) project, a German follow-up project on an international survey study (COVID-19 International Student Well-being Study, C19 ISWS) that was conducted in the beginning of the COVID-19 pandemic in various countries (Van de Velde et al., 2021). The C19 GSWS was carried out to examine the health and health (risk) behaviours among university students in Germany at a later stage of the pandemic (approx. 20 months after instalment of first COVID-19 measures in Germany).

### Study Population

For the study, a convenience sampling strategy was employed at five German universities (Charité – Universitätsmedizin Berlin, University of Bremen, Heinrich-Heine-University Düsseldorf, Martin—Luther University Halle-Wittenberg, University of Siegen). University students were recruited to the study by emails, adverts on virtual learning environments and social media. To participate, students had to be at least 18 years old, enrolled at one of the five universities, and registered in an undergraduate, graduate, state examination, or doctoral program.

### Data Collection

The data collection was conducted between October 27th and November 14th 2021. At this time, Germany experienced the fourth COVID-19 wave and the COVID-19 incidence increased a fourth time, driven by the spread of the SARS-CoV-2 delta variant. During the survey period, several COVID-19 regulations, such as the obligation of wearing masks indoors or hygiene measures, were still in place and were also mandatory at the participating higher education institutions. For teaching purposes, most German universities used a remote format and only a fraction of classes were conducted in person or in a hybrid format mostly in smaller working groups and under strict health guidelines. The learn and teaching environment therefore differed substantially from the normal situation at all participating universities.

Students were asked to fill out an anonymous online survey that was available in English and German on LimeSurvey. Participating was voluntary and students gave their informed consent before participating in the survey. The ethics committees of the five universities granted ethical approval for the study (University of Bremen 2021-28-EIL; University Halle-Wittenberg 2020-066; Heinrich-Heine-University Duesseldorf 2020-958_1; Charité – Universitätsmedizin Berlin; and University of Siegen).

### Questionnaire

The questionnaire used in this study was based the C19 ISWS. The development of the survey and the selected instruments were described elsewhere (Van de Velde et al., 2021). The survey included questions on the student’s socio-demographic characteristics, study-related information, their personal SU and PA, as well as their mental well-being during the pandemic. Moreover, COVID-19 vaccination status, and attitudes towards COVID-19 vaccinations were assessed. The original C19 ISWS questionnaire is available open access (https://zenodo.org/records/3928457).

### Outcomes

To measure SU, students were asked about their (1) smoking (tobacco and e-cigarette use), (2) binge drinking, (3) cannabis use during the last week. To measure PA, (4) engagement in vigorous physical activity (VPA) and (5) moderate physical activity (MPA) during the last week prior to filling out the survey was assessed. Smoking and e-cigarette use were assessed by the question ‘How often in the last week did you smoke tobacco (cigarettes, cigars, e-cigarettes/vaporizers)?’. To estimate binge drinking occasions, students were asked ‘How often did you drink six or more glasses of alcohol on a single occasion during the last week?’. Cannabis use was assessed as follows: ‘During the last week, how often did you use cannabis (marijuana, weed, hash…)?’. VPA was assessed by the question ‘During the last week, how often did you perform vigorous physical activities like lifting heavy things, running, aerobics, or fast cycling for at least 30 min?’ while for MPA the question ‘During the last week, how often did you perform moderate physical activities like easy cycling or walking for at least 30 min?’ was asked.

Response options on a 5-point scale were ‘(almost) never’, ‘less than once a week’, ‘once per week’, ‘more than once per week’ and ‘(almost) daily’. Participants who reported smoking, binge drinking, and cannabis use ‘once per week or more’ were considered harmful and compared to ‘never or less than once per week’. Engaging in moderate or vigorous PA ‘(almost) never or less than once a week’ (low PA) was compared to engagement ‘once per week or more’ (high PA).

### Covariates

Sociodemographic data, study-related information, depressive symptoms, having a trusted person and boredom were considered as covariates. As sociodemographic data, age was used as a continuous variable, whereas gender (female/male/diverse), place of birth (born in Germany/not born in Germany) and relationship status (single/in a relationship/’it’s complicated’) were used in a categorized manner. Study-related information was included as follows: Being a first-year student (yes/no), which study subject they were enrolled in (health sciences or medicine/other study fields), and how important studies are compared to other activities (e.g., meeting with friends, doing hobbies, etc.) (more important/equally important/less important). Depressive symptoms were measured using the 8-item Centre for Epidemiologic Studies—Depression Scale (CES-D 8). In the CES-D 8, respondents were asked how often in the previous week they felt ‘depressed’, ‘lonely’, ‘sad’, ‘happy’, ‘enjoyed life’, ‘felt every-thing they did was an effort’, had ‘restless sleep’ and ‘could not get going’. Response options on the 4-point Likert scale ranged from (0) ‘none or almost none of the time’, (1) ‘sometimes’, (2) ‘most of the time’ to (3) ‘all or almost all of the time’. The two items ‘happy’ and ‘enjoyed life’ were reverse coded and scores were summed up. The CES-D 8 scale was used as a continuous variable. The full version of the CES-D has been shown to be reliable and validated for use within the university setting (Jiang et al., 2019) The abbreviated version of the CES-D with eight items is also frequently used in this population, as well as in the previous C19 ISWS (Van de Velde et al., 2021). The short form was validated in a sample of university students in Czechia during the pandemic (Klusáček et al., 2022) and provides an efficient means of screening for depressive symptoms and is preferred in large-scale research due to its convenience. The Cronbach’s alpha in our sample was 0.86 for CES-D 8. Availability of a trusted person was assessed by the question ‘Do you have anyone with whom you can discuss any intimate and personal matters?’ (yes/no). Regarding boredom, students were asked to indicate how much of the time during the past week they were bored (never or sometimes/most of the times or always).

### Statistical Analysis

We conducted a complete case analysis with respect to each outcome. Descriptive analysis was performed using tabulations for each health behaviour. For each outcome one multivariable logistic regression analysis was conducted to examine associations between each SU and PA variable and the independent variables. In order to identify profiles of considered health behaviours in participants, similar to the approach of identifying drinking patterns presented by Aresi et al. (2022), a latent class analysis (LCA) was conducted considering two to five latent profiles of five health behaviour variables. The different LCAs were compared by considering the Analytic Hierarchy Process (AHP) including multiple fit statistics (Akogul & Erisoglu, 2017; Zhang et al., 2018). Differences in CESD-8 by strata of the chosen profiles were evaluated in terms of boxplots. Descriptive statistics and regressions were conducted in SPSS Version 26.0. We used the poLCA (version 1.6.0.1 (Linzer & Lewis, 2011)) in R 4.3.3 to conduct the latent class analysis for categorical variables.

## Results

The web-based questionnaire included information by 7203 university students enrolled at five institutions. On average, participants were 24.1 years (SD = 4.9) old, 67.9% were females and 31.0% males. The majority of participants were born in Germany (91.4%) and were in a relationship (52.7%). Table 1 presents the characteristics of the study sample.

**Table 1:** Characteristics of C19 GSWS study sample

	Variables	Mean (± SD)/n (%)
Socio-demographic information	Age (n = 7181)	24.1 (4.9)
	Gender (n = 7100)	
	Female	4824 (67.9)
	Male	2199 (31.0)
	Diverse	77 (1.1)
	Place of birth (n = 7158)	
	In Germany	6539 (91.4)
	Outside Germany	619 (8.6)
	Relationship status (n = 7062)	
	Single	2963 (42.0)
	In a relationship	3797 (52.7)
	It’s complicated	302 (4.3)
Study-related information	Universities (n = 7203)	
	Charité- Universitätsmedizin Berlin	1,131 (15.7)
	University of Bremen	1819 (25.3)
	Heinrich-Heine University Düsseldorf	520 (7.2)
	Martin–Luther University Halle	2168 (30.1)
	University of Siegen	1565 (21.7)
	First-year student (n = 7173)	1404 (19.5)
	Health-related study field (n = 7177)	1905 (26.4)
	Importance of studying compared to other activities (n = 7197)	
	More important	2577 (35.8)
	Equally important	4069 (56.5)
	Less important	551 (7.6)
Further covariates	Availability of trusted person (n = 6,289)	
	Yes	5.688 (90.4)
	No	601 (9.6)
	Feeling bored (n = 6915)	
	Never/sometimes	5959 (86.2)
	Most of the times/always	956 (13.8)
	Depressive symptoms (n = 6848)	9.4 (4.9)

### Rates of Substance Use and Physical Activity (RQ1)

16.5% of participants reported smoking, 19.2% binge drinking and 6.4% cannabis use at least once during the last week. Low PA was reported by 13,4% with regard to MPA and 35.0% with regard to VPA (Table 2).

**Table 2:** Substance use and physical activity among German university students (in %)

	Smoking (n = 7073)	Binge drinking (n = 7097)	Cannabis use (n = 7062)	Vigorous physical activity (n = 7127)	Moderate physical activity (n = 7163)
(Almost) none / never or less than once a week	5906 (83.5)	5736 (80.8)	6609 (93.6)	2494 (35.0)	957 (13.4)
Once a week or daily	1167 (16.5)	1361 (19.2)	453 (6.4)	4633 (65.0)	6206 (86.6)

### Associated Factors with Engagement in SU and PA (RQ2)

In the regression model, being male was found to be associated with a higher chance of reporting to smoke cigarettes, e-cigarettes/vaporizers or cigars (OR 1.57, 95% CI 1.35–1.83), to engage in binge drinking (OR 2.17, 95% CI 1.89–2.49) or to use cannabis at least once per week (OR 2.15, 95% CI 1.71–2.69). Male students had a reduced chance (OR 0.78, 95% CI 0.69–0.88) for low vigorous physical activity compared to female students. Being in a complicated relationship was associated with smoking (OR 1.91, 95% CI 1.42–2.56), binge drinking (OR 1.57, 95% CI 1.16–2.13), and cannabis use (OR 1.68, 95% CI 1.12–2.54), but not with low levels of PA compared to being in a steady relationship. Studying a health-related study field, such as health sciences or medicine, showed a protective association with smoking and cannabis use and being engaged in lower levels of VPA or MPA (Table 3).

**Table 3:** Associations between substance use, physical activity and age, gender, place of birth, relationship status, being a first-year student, study field, importance of studies, trusted other, boredom as well as depressive symptoms – Results of multivariable binary logistic regressions (ORs and 95%-CI)

		Smoking at least once per week (n = 5868)		Binge drinking at least once per week (n = 5891)		Cannabis use at least once per week (n = 5863)		Vigorous PA less than once per week (n = 5908)		Moderate PA less than once per week (n = 5939)	
		OR	95%-CI	OR	95%-CI	OR	95%-CI	OR	95%-CI	OR	95%-CI
Age (per 1 year)		1.04	1.03–1.06	0.97	0.96–0.99	1.00	0.98–1.03	1.01	1.00–1.02	1.00	0.98–1.01
Gender	Female (ref = 1)										
	Male	1.57	1.35–1.83	2.17	1.89–2.49	2.15	1.71–2.69	0.78	0.69–0.88	1.07	0.90–1.27
	Diverse	1.58	0.77–2.85	0.51	0.20–1.28	1.40	0.50–3.95	0.95	0.55–1.64	0.49	0.20–1.24
Place of birth	Ger-many (ref = 1)										
	Other	1.35	1.06–1.74	0.72	0.55–0.96	1.32	0.90–1.92	1.26	1.03–1.54	1.29	0.99–1.68
Relationship status	Relation-ship (ref = 1)										
	Single	0.84	0.72–0.98	1.04	0.90–1.20	0.63	0.49–0.80	0.98	0.87–1.10	0.90	0.76–1.06
	Complicated	1.91	1.42–2.56	1.57	1.16–2.13	1.68	1.12–2.54	0.84	0.64–1.11	1.14	0.80–1.62
First-year student	No (ref = 1)										
	Yes	1.03	0.85–1.25	0.88	0.74–1.05	1.01	0.75–1.34	1.28	1.11–1.48	1.07	0.87–1.30
Health -related study field	No (ref = 1)										
	Yes	0.68	0.56–0.81	1.07	0.91–1.24	0.73	0.56–0.97	0.79	0.70–0.90	0.70	0.57–0.85
Importance of studies compared to other activities	More import-ant (ref = 1)										
	Equally important	1.42	1.21–1.67	1.63	1.40–1.89	1.87	1.44–2.42	0.88	0.78–0.99	0.81	0.69–0.95
	Less important	2.28	1.77–2.94	2.29	1.79–2.93	3.31	2.29–4.76	1.04	0.84–1.29	0.70	0.50–1.00
Having a trusted other	Yes (ref = 1)										
	No	0.81	0.63–1.05	0.76	0.59–0.98	0.88	0.60–1.29	1.20	0.99–1.45	1.37	1.08–1.74
Being bored	Never/ some-times (ref = 1)										
	Most of the times/always	0.93	0.75–1.15	0.95	0.77–1.17	1.08	0.81–1.47	1.36	1.15–1.61	1.45	1.18–1.78
Depressive symptoms (per one unit on CESD-8 scale)		1.07	1.06–1.09	1.02	1.00–1.03	1.07	1.04–1.09	1.06	1.05–1.07	1.09	1.07–1.11

Reporting depressive symptoms was associated with smoking, cannabis use, and low levels of MPA/VPA during the last week but not with binge drinking.

Students who reported that they found their studies less or equally important as other activities showed an increased chance for weekly engagement in smoking, binge drinking, and cannabis use compared to students who found their studies more important than other activities. Finding studying equally or less important compared to other activities was associated with lower odds for performing lower levels of VPA and MPA (Table 3).

Additionally, students who reported being bored most or all of the time were more likely to report more often vigorous (OR 1.36, 95% CI 1.15–1.61) and moderate (OR 1.45, 95% CI 1.18–1.78) PA less than once per week compared to students being never or sometimes bored (Table 3).

### Profiles of Health Behaviours (RQ3)

Results of the latent class analysis are presented in the supplemental Table S1 and S2. Due to item missings in any of the five health behavior variables, LCA was only conducted in 6628 participants. Table S1 comprises multiple fit statistics comparing two to five latent classes. The majority of the fit indices (e.g. AIC, CAIC, BIC, ssBIC) indicated the best fit with the four-class model together with a significant LMRT statistic. Conditional probabilities of the four-class model are presented in Table S2. Most of the profiles indicated high PA in participants and higher probabilities for no / low drug use with some differences in the probabilties for licit and illicit substance use. For characterization the profiles were named: (1) health-protective behavior, (2) moderate PA and smoking, (3) licit and illicit substance use, (4) moderate PA and no / low drug use.

**Fig. 1:**
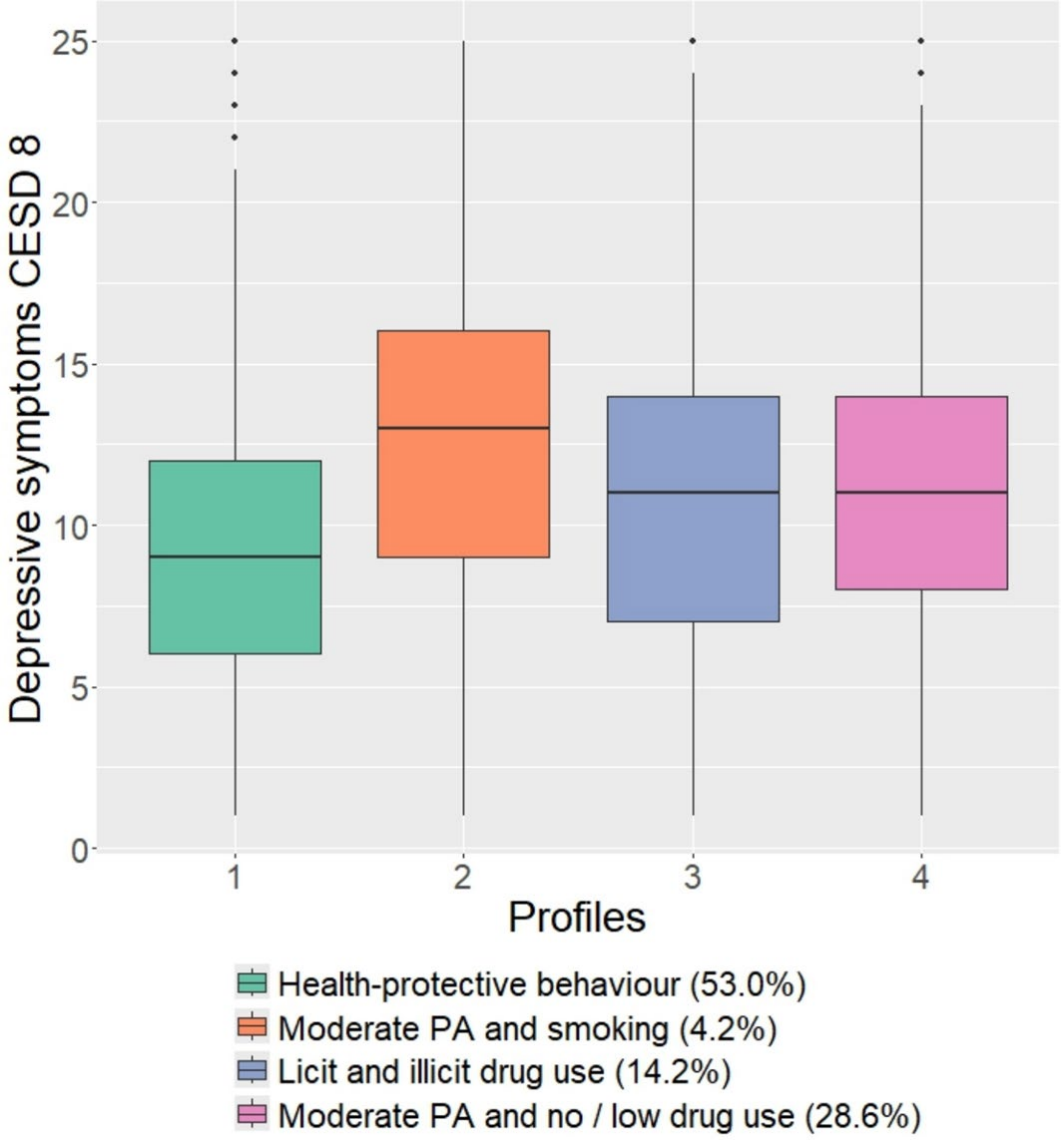
Boxplots of depressive symptoms measured by CESD-8 stratified by substance use and physical activity profiles

These profiles mainly differed with regard to VPA and MPA. The highest percentage of participants (53.0%) fell into the profile ‘health-protective behaviour’ with high overall physical activity (once per week or more) and generally low to no substance use. 28.6% of respondents fell into the profile ‘moderate PA’ with moderate physical activity and low to no drug use. In addition, 4.2% of respondents reported moderate physical activity and higher item probabilities for current smoking and were subsumed under the profile ‘moderate PA and smoking’. A fourth profile ‘licit and illicit substance use’ combined higher proportions of other substance use together with a high probability for vigorous PA and was found in 14.2% of the study population (supplementary material, Table S2). Boxplots of CESD-8 by profiles revealed lowest CESD-8 scores in the health-protective behavior profile and highest CESD-8 scores in students associated with the smoking and moderate physical activity profile (Fig. 1).

## Discussion

This study investigated the prevalence of SU and PA, factors associated with SU and PA, as well as profiles of health behaviours, in university students in Germany 20 months into the COVID-19 pandemic.

Low physical activity and binge drinking were found to be the most reported health risk behaviours (RQ1). We found that depressive symptoms, being bored and study-related factors were found to be associated with SU and PA (RQ2). Moreover, most students showed clustering of health protective behaviours, followed by low physical activity with low substance use (RQ3).

Generally, evidence suggests, that the COVID-19 pandemic led to substantial changes in health behaviours (Acuff et al., 2022; Tavolacci et al., 2021). For instance, a scoping review of 129 studies on cannabis use during the COVID-19 pandemic found that nearly half of the studies reported an increase or initiation in cannabis use during the pandemic (Mehra et al., 2023). Comparing SU and PA percentages revealed in this study with a similar study based on a sample recruited at almost the same universities from May 2020 shows that binge drinking (25% in the beginning of the pandemic) and cannabis use (9% in the beginning of the pandemic) seemed to have declined during the course of the pandemic, while smoking stayed at the same level (Busse et al., 2021). This is not in line with findings from another study conducted among students at one German university in 2020 and 2021, which reported increasing alcohol and drug consumption, however, different measurements for alcohol and drug consumption were used in the survey (Dogan-Sander et al., 2021). Regarding engagement in physical activity, the present study found that 13% university students reported low levels of moderate and 35% of vigorous physical activity, presenting lower inactivity levels compared to the 44% of students found to be inactive in a survey from May 2020 (Busse et al., 2021). A comparative analysis of cross-sectional data from spring and autumn of 2020 among university students in Switzerland reported less physical activity later on in the pandemic than in the beginning of the pandemic (Volken et al., 2021).

Inconclusiveness in changes in health behaviour, such as alcohol consumption during the pandemic may be explained by different mechanisms: On one hand, the pandemic inducing increasing psychological stressors (fewer financial resources, isolation from others, uncertainty about the future) may have led to the uptake of unhealthy behaviours as coping strategies. On the other hand, the COVID-19-related restrictions may have led to reduced opportunities to engage in risky behaviours, such as alcohol use at social gatherings, but also to engage in healthy behaviours such as sport clubs or gyms (Rehm et al., 2020).

We found that the presence of depressive symptoms was associated with reporting all studied health behaviours except for binge drinking. This finding related to the existing literature which has shown the close link between SU and PA and mental health, also prior to the pandemic (Atienza-Carbonell et al., 2022; Busse et al., 2021; Giner-Murillo et al., 2021; Kenney et al., 2018; Lechner et al., 2020). In addition to the factors considered in our study, there are also other influences contributing to engagement in health behaviours, e.g., self-isolation which was initially recommended by public authorities as a public health measure during the pandemic (Bartel et al., 2020). We further found that students who reported being bored most or all of the time were more prone to reporting infrequent vigorous and moderate PA compared to those who experienced boredom less frequently. This is also in line with previous findings that have established the relation between boredom and engagement in PA in university students in Germany (Bösselmann et al., 2021).

Study-related factors were also found in the present study to be associated with SU and PA. Students enrolled in health-related study fields were less likely to engage in smoking and cannabis use or to be physically inactive. Those who perceived their studies as less or equally important compared to other activities were found to have a higher likelihood for engaging in weekly smoking, binge drinking, and cannabis use, in contrast to students who prioritized their studies above other activities.

To our knowledge, this is the first study analyzing this association; however, studies conducted in the school setting showed similar findings, that school connectedness is associated with lower substance use and high PA levels (Weatherson et al., 2018).

In our sample, positive health behaviours clustered, with the majority of students belonging to the profile that showed high levels of physical activity and no or low levels of substance use. Similarly, other research found that most students reported a health-protective lifestyle (van Hooijdonk et al., 2023). An analysis of alcohol use patterns in young Italian adults between the ages of 18 and 34 years revealed that non-risky drinking behaviour was most prevalent, but in comparison with the time prior to the pandemic, this pattern decreased, whereas current non-drinking and weekly risky drinking increased (Aresi et al., 2022). Profiles found in our analysis did not find higher probabilities of low physical activity in combination with binge drinking and cannabis, but rather of low physical activity with smoking, which aligns with other studies (Bennasar-Veny et al., 2020; van Hooijdonk et al., 2023). These findings can inform future prevention campaigns. Even though prevalence of specific health behaviours such as binge drinking is high among students, we did not identify a profile of students showing risky levels of all analysed health behaviours. However, smoking and low physical activity were found as a profile in 6.4 of the students, so that specific programmes addressing SU as well as PA during the pandemic can be of interest (Pfledderer et al., 2022).

### Strengths and Limitations

Due to relying on self-reported data, it is possible that SU/PA were either under—or overreported. To mitigate this potential distortion, our data were collected via a confidential online survey. Even though more than 10% of enrolled students at most sites participated in the study, we could not ensure representative participation. A high proportion of participants studied medicine or health-related subjects, and we found a gender imbalance, which limits the generalisability of the results to the entire university student population. Therefore, the observed SU/PA percentages may not be representative for university students in Germany or even in the participating universities. While the present study was based on data assessed in the C19 ISWS project with a similar survey conducted in 2020, and the invitation was of students was conducted in the same way, the participants were not necessarily identical, and the study design did not allow linkage of data at the individual level. Additionally, our cross-sectional analysis does not allow for causal inferences. For the questions on SU and PA, the reference period of one week was used, which was done due to the survey period with many changes in regulations and in order to ensure comparability with the initial study, in which the same questions were used to assess current behaviour. To represent problem SU, binge drinking in the last week was assessed. The threshold of six drinks was chosen, which is also used in the AUDIT-C (Bush et al., 1998). In this survey, no gender-specific questions were used, as the empirical evidence for this was found to be insufficient in other studies among college students (Pearson et al., 2016). However, the uniform threshold may result in a loss of information, particularly regarding female problem drinking. In our study, we did not address potential interactions between variables, as this was our first exploration of influencing factors at universities in a later stage of the pandemic. Future studies could explore the potential influencing factors in more detail and focus on interactions. Another limitation of our study is the limited comparability with the literature, as most studies refer to time points shortly after the start of the pandemic, whereas our study examines a later stage (20 months after the start) of the pandemic. Therefore, comparisons between our study and the literature may have limited validity.

Despite these limitations, certain strengths also warrant mentioning. While the majority of COVID-19 studies on health behaviours focussed on the early period of the pandemic following the implementation of measures to contain the spread of the virus, this study was conducted 20 months following the first implementation of measures. With that in mind, this study provides a unique snapshot of SU and PA as well as their correlates in university students in Germany at a stage of the pandemic where the student population was already used to structural changes due to the pandemic such as the switch to online teaching.

### Implications for Research and Prevention Practice

From a public health perspective, further research into the influence of crises situations on specific health behaviours is of high importance. While crises might offset current healthy behaviours, substantial life events have also been shown to positively influence health behaviours and might lead to sustained positive behaviour change (Ogden & Hills, 2008). Understanding the mechanisms by which life events and transitions impact on SU/PA and how they foster either positive and negative change will be an interesting avenue to explore in future research. Moreover, the associations between high-risk health behaviours and depressive symptoms warrant further attention and suggest a need for longitudinal studies to explore causal relationships.

As several factors seem to be associated with health behaviours, future interventions addressing these should be designed and tailored to different user groups, particularly those at highest risk. Participatory approaches in intervention development show promise in ensuring that intervention activities take into account the perspective and needs of the target group and are designed in a way that is acceptable and feasible (Wright et al., 2016). In a similar line, evaluations of interventions aimed at SU/PA should involve subgroup analyses to ensure that the targeted user groups actually participate in the study and equally benefit from the intervention (Krukowski et al., 2024).

The link between depressive symptoms, as well as academic involvement and SU/PA, underlines the importance of integrating mental health services and counselling services within the university. Moving beyond merely implementing singular health-promoting interventions, universities should adhere to the Health Promoting Universities approach and apply a settings approach to health (Dooris & Doherty, 2010; Stock et al., 2022).

## Conclusion

Twenty months into the pandemic, several factors were shown to be associated with substance use and physical inactivity such as reporting depressive symptoms and study-related factors. Profiles of health risk behaviours could be identified, and it was found that most students showed ‘health-protective behaviour’ or low physical activity. These findings are particularly relevant for health promotion and prevention in university settings and indicate potential benefits of tailored interventions not only focussing on reducing substance use and increasing physical activity, but also on mental health.

## Supplementary Information

Below is the link to the electronic supplementary material.

Supplementary file1 (DOCX 24 KB)

## References

[CR1] Acuff, S. F., Strickland, J. C., Tucker, J. A., & Murphy, J. G. (2022). Changes in alcohol use during COVID-19 and associations with contextual and individual difference variables: A systematic review and meta-analysis. *Psychology of Addictive Behaviors,**36*(1), 1–19. 10.1037/adb0000796 34807630 10.1037/adb0000796 PMC8831454

[CR2] Akogul, S., & Erisoglu, M. (2017). An approach for determining the number of clusters in a model-based cluster analysis. *Entropy,**19*(9), 452.

[CR3] Aresi, G., Sorgente, A., Cleveland, M. J., & Marta, E. (2022). Patterns of alcohol use among Italian young adults before and during a COVID-19 Lockdown: A latent class analysis study. *Journal of Prevention,**43*(2), 191–208. 10.1007/s10935-022-00675-2 35305212 10.1007/s10935-022-00675-2 PMC8934024

[CR4] Atienza-Carbonell, B., Guillén, V., Irigoyen-Otiñano, M., & Balanzá-Martínez, V. (2022). Screening of substance use and mental health problems among Spanish medical students: A multicenter study. *Journal of Affective Disorders,**311*, 391–398. 10.1016/j.jad.2022.05.090 35609765 10.1016/j.jad.2022.05.090

[CR5] Bartel, S. J., Sherry, S. B., & Stewart, S. H. (2020). Self-isolation: A significant contributor to cannabis use during the COVID-19 pandemic. *Substance Abuse,**41*(4), 409–412. 10.1080/08897077.2020.1823550 33044893 10.1080/08897077.2020.1823550

[CR6] Bennasar-Veny, M., Yañez, A. M., Pericas, J., Ballester, L., Fernandez-Dominguez, J. C., Tauler, P., & Aguilo, A. (2020). Cluster analysis of health-related lifestyles in university students. *International Journal of Environmental Research and Public Health*. 10.3390/ijerph17051776 32182922 10.3390/ijerph17051776 PMC7084566

[CR7] Bösselmann, V., Amatriain-Fernández, S., Gronwald, T., Murillo-Rodríguez, E., Machado, S., & Budde, H. (2021). Physical activity, boredom and fear of COVID-19 among adolescents in Germany. *Frontiers in Psychology*. 10.3389/fpsyg.2021.624206 34012413 10.3389/fpsyg.2021.624206 PMC8126672

[CR8] Botella-Juan, L., Amezcua-Prieto, C., Morales-Suarez-Varela, M. M., Mateos-Campos, R., Ayán-Pérez, C., Molina, A. J., Ortiz-Moncada, R., Redondo-Martín, S., Alguacil, J., Blázquez-Abellán, G., Delgado-Rodríguez, M., Alonso-Molero, J., & Fernández-Villa, T. (2022). Impact of the COVID-19 pandemic on the evolution of prevalence and patterns of cannabis use among first-year university students in Spain—UniHcos project. *International Journal of Environmental Research and Public Health,**19*(18), 11577. 36141846 10.3390/ijerph191811577 PMC9517240

[CR9] Botella-Juan, L., Morales-Suárez-Varela, M., Amezcua-Prieto, C., Mateos-Campos, R., Ayán-Pérez, C., Molina, A. J., Ortiz-Moncada, R., Delgado-Parrilla, A., Blázquez-Abellán, G., Delgado-Rodríguez, M., Alonso-Molero, J., & Fernández-Villa, T. (2024). Changes in alcohol consumption during the COVID-19 among first-year university students in Spain, considering the risk of problematic use - UniHcos project. *Alcohol,**120*, 133–141. 10.1016/j.alcohol.2024.06.008 38969249 10.1016/j.alcohol.2024.06.008

[CR10] Brooks, S. K., Webster, R. K., Smith, L. E., Woodland, L., Wessely, S., Greenberg, N., & Rubin, G. J. (2020). The psychological impact of quarantine and how to reduce it: Rapid review of the evidence. *The Lancet,**395*(10227), 912–920. 10.1016/S0140-6736(20)30460-8 10.1016/S0140-6736(20)30460-8 PMC7158942 32112714

[CR11] Bush, K., Kivlahan, D. R., McDonell, M. B., Fihn, S. D., & Bradley, K. A. (1998). The AUDIT alcohol consumption questions (AUDIT-C): An effective brief screening test for problem drinking ambulatory care quality improvement project (ACQUIP) alcohol use disorders identification test. *Archives of Internal Medicine,**158*(16), 1789–1795. 10.1001/archinte.158.16.1789 9738608 10.1001/archinte.158.16.1789

[CR12] Busse, H., Buck, C., Stock, C., Zeeb, H., Pischke, C. R., Fialho, P. M. M., Wendt, C., & Helmer, S. M. (2021). Engagement in health risk behaviours before and during the COVID-19 pandemic in german university students: Results of a cross-sectional study. *International Journal of Environmental Research and Public Health*. 10.3390/ijerph18041410 33546344 10.3390/ijerph18041410 PMC7913592

[CR13] Castro, O., Bennie, J., Vergeer, I., Bosselut, G., & Biddle, S. J. H. (2020). How sedentary are university students? A systematic review and meta-analysis. *Prevention Science,**21*(3), 332–343. 10.1007/s11121-020-01093-8 31975312 10.1007/s11121-020-01093-8

[CR14] Davoren, M. P., Demant, J., Shiely, F., & Perry, I. J. (2016). Alcohol consumption among university students in Ireland and the United Kingdom from 2002 to 2014: A systematic review. *BMC Public Health,**16*(1), 173. 10.1186/s12889-016-2843-1 26895824 10.1186/s12889-016-2843-1 PMC4759952

[CR15] Dogan-Sander, E., Kohls, E., Baldofski, S., & Rummel-Kluge, C. (2021). More depressive symptoms, alcohol and drug consumption: Increase in mental health symptoms among university students after one year of the COVID-19 pandemic. *Frontiers in Psychiatry,**12*, Article 790974. 10.3389/fpsyt.2021.790974 34975589 10.3389/fpsyt.2021.790974 PMC8716753

[CR50] Dooris, M., & Doherty, S. (2010). Healthy universities — time for action: a qualitative research study exploring the potential for a national programme. *Health Promotion International*, **25**(1), 94–106. 10.1093/heapro/daq015 10.1093/heapro/daq015 20167825

[CR16] Gewalt, S. C., Berger, S., Krisam, R., & Breuer, M. (2022). Effects of the COVID-19 pandemic on university students’ physical health, mental health and learning, a cross-sectional study including 917 students from eight universities in Germany. *PLoS ONE,**17*(8), Article e0273928. 10.1371/journal.pone.0273928 36044521 10.1371/journal.pone.0273928 PMC9432688

[CR17] Giner-Murillo, M., Atienza-Carbonell, B., Cervera-Martínez, J., Bobes-Bascarán, T., Crespo-Facorro, B., De Boni, R. B., Esteban, C., García-Portilla, M. P., Gomes-da-Costa, S., González-Pinto, A., Jaén-Moreno, M. J., Kapczinski, F., Ponce-Mora, A., Sarramea, F., Tabarés-Seisdedos, R., Vieta, E., Zorrilla, I., & Balanzá-Martínez, V. (2021). Lifestyle in undergraduate students and demographically matched controls during the COVID-19 pandemic in Spain. *International Journal of Environmental Research and Public Health,**18*(15), 8133. 34360426 10.3390/ijerph18158133 PMC8346054

[CR18] Goncalves, A., Le Vigouroux, S., & Charbonnier, E. (2021). University students’ lifestyle behaviors during the COVID-19 pandemic: A four-wave longitudinal survey. *International Journal of Environmental Research and Public Health,**18*(17), 8998. 34501605 10.3390/ijerph18178998 PMC8430950

[CR19] Heradstveit, O., Sivertsen, B., Lønning, K.-J., & Skogen, J. C. (2022). The extent of alcohol-related problems among college and university students in Norway prior to and during the COVID-19 pandemic. *Frontiers in Public Health*. 10.3389/fpubh.2022.876841 35719681 10.3389/fpubh.2022.876841 PMC9204355

[CR20] Jiang, L., Wang, Y., Zhang, Y., Li, R., Wu, H., Li, C., Wu, Y., & Tao, Q. (2019). The reliability and validity of the center for epidemiologic studies depression scale (CES-D) for Chinese University students. *Front Psychiatry,**10*, 315. 10.3389/fpsyt.2019.00315 31178764 10.3389/fpsyt.2019.00315 PMC6537885

[CR21] Kale, D., Perski, O., Herbec, A., Beard, E., & Shahab, L. (2022). Changes in cigarette smoking and vaping in response to the COVID-19 pandemic in the UK: Findings from baseline and 12-month follow up of HEBECO study. *International Journal of Environmental Research and Public Health*. 10.3390/ijerph19020630 35055451 10.3390/ijerph19020630 PMC8775930

[CR22] Kenney, S. R., DiGuiseppi, G. T., Meisel, M. K., Balestrieri, S. G., & Barnett, N. P. (2018). Poor mental health, peer drinking norms, and alcohol risk in a social network of first-year college students. *Addictive Behaviors,**84*, 151–159. 10.1016/j.addbeh.2018.04.012 29684764 10.1016/j.addbeh.2018.04.012 PMC5975188

[CR23] Klusáček, J., Kudrnáčová, M., & Soukup, P. (2022). Validation of CES-D8 among Czech university students during COVID-19 pandemic. *Československá Psychologie: Časopis Pro Psychologickou Teorii a Praxi,**66*(4), 398–415. 10.51561/cspsych.66.4.398

[CR49] Krukowski, R. A., Ross, K. M., Western, M. J., Cooper, R., Busse, H., Forbes, C., Kuntsche, E., Allmeta, A., Macedo Silva, A., John-Akinola, Y.O., & König, L. M. (2024). Digital health interventions for all? Examining inclusivity across all stages of the digital health intervention research process. *Trials*, **25**(1), 98. 10.1186/s13063-024-07937-w 10.1186/s13063-024-07937-w PMC10826214 38291539

[CR24] Larsson, K., Onell, C., Edlund, K., Källberg, H., Holm, L. W., Sundberg, T., & Skillgate, E. (2022). Lifestyle behaviors in Swedish university students before and during the first six months of the COVID-19 pandemic: A cohort study. *BMC Public Health,**22*(1), 1207. 10.1186/s12889-022-13553-7 35710368 10.1186/s12889-022-13553-7 PMC9202972

[CR25] Lechner, W. V., Laurene, K. R., Patel, S., Anderson, M., Grega, C., & Kenne, D. R. (2020). Changes in alcohol use as a function of psychological distress and social support following COVID-19 related University closings. *Addictive Behaviors,**110*, Article 106527. 10.1016/j.addbeh.2020.106527 32679435 10.1016/j.addbeh.2020.106527 PMC7319610

[CR26] Linzer, D. A., & Lewis, J. B. (2011). poLCA: An R package for polytomous variable latent class analysis. *Journal of Statistical Software,**42*(10), 1–29. 10.18637/jss.v042.i10

[CR27] López-Valenciano, A., Suárez-Iglesias, D., Sanchez-Lastra, M. A., & Ayán, C. (2020). Impact of COVID-19 pandemic on university students’ physical activity levels: An early systematic review. *Frontiers in Psychology,**11*, Article 624567. 10.3389/fpsyg.2020.624567 33519653 10.3389/fpsyg.2020.624567 PMC7845570

[CR28] Mehra, K., Rup, J., Wiese, J. L., Watson, T. M., Bonato, S., & Rueda, S. (2023). Changes in self-reported cannabis use during the COVID-19 pandemic: A scoping review. *BMC Public Health,**23*(1), 2139. 10.1186/s12889-023-17068-7 37915021 10.1186/s12889-023-17068-7 PMC10621278

[CR52] Ogden, J., & Hills, L. (2008). Understanding sustained behavior change: the role of life crises and the process of reinvention. *Health*, *12*(4), 419-437. 10.1177/1363459308094417 10.1177/1363459308094417 18818273

[CR29] Pearson, M. R., Kirouac, M., & Witkiewitz, K. (2016). Questioning the validity of the 4+/5+ binge or heavy drinking criterion in college and clinical populations. *Addiction,**111*(10), 1720–1726. 10.1111/add.13210 27605077 10.1111/add.13210 PMC5017312

[CR30] Pfledderer, C. D., Bai, Y., Brusseau, T. A., Burns, R. D., & King Jensen, J. L. (2022). Changes in college students’ health behaviors and substance use after a brief wellness intervention during COVID-19. *Preventive Medicine Reports,**26*, 101743. 10.1016/j.pmedr.2022.101743 35242504 10.1016/j.pmedr.2022.101743 PMC8885082

[CR31] Rehm, J., Kilian, C., Ferreira-Borges, C., Jernigan, D., Monteiro, M., Parry, C. D. H., Sanchez, Z. M., & Manthey, J. (2020). Alcohol use in times of the COVID 19: Implications for monitoring and policy. *Drug and Alcohol Review,**39*(4), 301–304. 10.1111/dar.13074 32358884 10.1111/dar.13074 PMC7267161

[CR32] Rivera, P. A., Nys, B. L., & Fiestas, F. (2021). Impact of COVID-19 induced lockdown on physical activity and sedentary behavior among university students: A systematic review. *Medwave,**21*(8), e8456. 10.5867/medwave.2021.08.8456 34487515 10.5867/medwave.2021.08.8456

[CR33] Rodríguez-Larrad, A., Mañas, A., Labayen, I., González-Gross, M., Espin, A., Aznar, S., Serrano-Sánchez, J. A., Vera-Garcia, F. J., González-Lamuño, D., Ara, I., Carrasco-Páez, L., Castro-Piñero, J., Gómez-Cabrera, M. C., Márquez, S., Tur, J. A., Gusi, N., Benito, P. J., Moliner-Urdiales, D., Ruiz, J. R., … Irazusta, J. (2021). Impact of COVID-19 confinement on physical activity and sedentary behaviour in spanish university students: Role of gender. *International Journal of Environmental Research and Public Health,**18*(2), 369. 33418907 10.3390/ijerph18020369 PMC7825050

[CR34] Romero-Blanco, C., Rodríguez-Almagro, J., Onieva-Zafra, M. D., Parra-Fernández, M. L., Prado-Laguna, M., & Hernández-Martínez, A. (2020). Physical activity and sedentary lifestyle in university students: Changes during confinement due to the COVID-19 pandemic. *International Journal of Environmental Research and Public Health,**17*(18), 6567. 32916972 10.3390/ijerph17186567 PMC7558021

[CR35] Rubio, M., van Hooijdonk, K., Luijten, M., Kappe, R., Cillessen, A. H. N., Verhagen, M., & Vink, J. M. (2023). University students’ (binge) drinking during COVID-19 lockdowns: An investigation of depression, social context, resilience, and changes in alcohol use. *Social Science & Medicine,**326*, 115925. 10.1016/j.socscimed.2023.115925 37137201 10.1016/j.socscimed.2023.115925 PMC10125214

[CR36] Savage, M. J., Hennis, P. J., Magistro, D., Donaldson, J., Healy, L. C., & James, R. M. (2021). Nine months into the COVID-19 pandemic: A longitudinal study showing mental health and movement behaviours are impaired in UK students. *International Journal of Environmental Research and Public Health,**18*(6), 2930. 33809313 10.3390/ijerph18062930 PMC7999965

[CR51] Stock, C. (2022). Grand challenges for public health education and promotion. *Frontiers in Public Health*, *10*, 917685. 10.3389/fpubh.2022.917685 35832282 10.3389/fpubh.2022.917685 PMC9271747

[CR37] Tavolacci, M. P., Wouters, E., Van de Velde, S., Buffel, V., Déchelotte, P., Van Hal, G., & Ladner, J. (2021). The impact of COVID-19 lockdown on health behaviors among students of a French University. *International Journal of Environmental Research and Public Health,**18*(8), 4346. 33923943 10.3390/ijerph18084346 PMC8072635

[CR38] Tholen, R., Ponnet, K., Van Hal, G., de Bruyn, S., Buffel, V., Van de Velde, S., Bracke, P., Bos, P., Akvardar, Y., Arnold, P., Busse, H., Chatzittofis, A., Helmer, S., Rabiee-Khan, F., Skalicka, V., Stathopoulou, T., Tavolacci, M. P., van der Heijde, C., & Wouters, E. (2024). Containment measures and alcohol consumption among drinking higher education students before and during the COVID-19 pandemic: A multilevel analysis in 25 countries. *Journal of Prevention*. 10.1007/s10935-024-00807-w 39325242 10.1007/s10935-024-00807-w

[CR39] Tholen, R., Ponnet, K., Van Hal, G., De Bruyn, S., Buffel, V., Van de Velde, S., Bracke, P., & Wouters, E. (2022). Substance use among belgian higher education students before and during the first wave of the COVID-19 pandemic. *International Journal of Environmental Research and Public Health*. 10.3390/ijerph19074348 35410029 10.3390/ijerph19074348 PMC8998911

[CR40] Van de Velde, S., Buffel, V., Bracke, P., Van Hal, G., Somogyi, N. M., Willems, B., & Wouters, E. (2021). The COVID-19 international student well-being study. *Scandinavian Journal of Public Health,**49*(1), 114–122. 10.1177/1403494820981186 33406995 10.1177/1403494820981186

[CR41] van Hooijdonk, K. J. M., Rubio, M., Simons, S. S. H., van Noorden, T. H. J., Luijten, M., Geurts, S. A. E., & Vink, J. M. (2022). Student-, study- and COVID-19-related predictors of students’ smoking, binge drinking and cannabis use before and during the initial COVID-19 lockdown in The Netherlands. *International Journal of Environmental Research and Public Health,**19*(2), 812. 35055634 10.3390/ijerph19020812 PMC8776226

[CR42] van Hooijdonk, K. J. M., Simons, S. S. H., van Noorden, T. H. J., Geurts, S. A. E., & Vink, J. M. (2023). Prevalence and clustering of health behaviours and the association with socio-demographics and mental well-being in Dutch university students. *Preventive Medicine Reports,**35*, 102307. 10.1016/j.pmedr.2023.102307 37519443 10.1016/j.pmedr.2023.102307 PMC10382923

[CR43] Vanderbruggen, N., Matthys, F., Van Laere, S., Zeeuws, D., Santermans, L., Van den Ameele, S., & Crunelle, C. L. (2020). Self-Reported alcohol, tobacco, and cannabis use during COVID-19 lockdown measures: Results from a web-based survey. *European Addiction Research,**26*(6), 309–315. 10.1159/000510822 32961535 10.1159/000510822 PMC7573904

[CR44] Volken, T., Zysset, A., Amendola, S., Klein Swormink, A., Huber, M., von Wyl, A., & Dratva, J. (2021). Depressive symptoms in swiss university students during the COVID-19 pandemic and its correlates. *International Journal of Environmental Research and Public Health*. 10.3390/ijerph18041458 33557193 10.3390/ijerph18041458 PMC7913894

[CR45] Weatherson, K. A., O’Neill, M., Lau, E. Y., Qian, W., Leatherdale, S. T., & Faulkner, G. E. J. (2018). The protective effects of school connectedness on substance use and physical activity. *Journal of Adolescent Health,**63*(6), 724–731. 10.1016/j.jadohealth.2018.07.002 10.1016/j.jadohealth.2018.07.002 30269908

[CR46] Wright, C. J. C., Dietze, P. M., Crockett, B., & Lim, M. S. C. (2016). Participatory development of MIDY (Mobile Intervention for Drinking in Young people). *BMC Public Health,**16*(1), 184. 10.1186/s12889-016-2876-5 26911299 10.1186/s12889-016-2876-5 PMC4765036

[CR47] Zhang, Z., Abarda, A., Contractor, A. A., Wang, J., & Dayton, C. M. (2018). Exploring heterogeneity in clinical trials with latent class analysis. *Annals of Translational Medicine,**6*(7), 119. 10.21037/atm.2018.01.24+ 29955579 10.21037/atm.2018.01.24 PMC6015948

[CR48] Zysset, A., Volken, T., Amendola, S., von Wyl, A., & Dratva, J. (2022). Change in alcohol consumption and binge drinking in university students during the early COVID-19 pandemic. *Frontiers in Public Health*. 10.3389/fpubh.2022.854350 35570889 10.3389/fpubh.2022.854350 PMC9092343

