# Supplementary material for: Factors Associated with Substance Use and Physical Activity Among German University Students 20 Months into the COVID-19 Pandemic
Source: J Prev (2022). 2025 Jul 5;46(6):933–51. doi: 10.1007/s10935-025-00865-8 (PMC12553565; doi:10.1007/s10935-025-00865-8)
Supplement: Supplementary file 1 — Supplementary file1 (DOCX 24 KB) [file 10935_2025_865_MOESM1_ESM.docx]

**Table S1.**

*Model fit statistics for Latent Class Analysis models with two to five classes in 6,628 participants.*

| **N class** | **Df** | **Gsq** | **χ2 LRT** | **-LL** | **HT** | **AIC** | **CAIC** | **AICc** | **BIC** | **ssBIC** | **HQ** | **LMR-LRT (p value)** |
| --- | --- | --- | --- | --- | --- | --- | --- | --- | --- | --- | --- | --- |
| **2** | 20 | 499.77 | 533.49 | -14,155.25 | 28332.54 | 28332.49 | 28418.28 | 28344.54 | 28407.28 | 28372.33 | 28358.33 | 862.165 (p <0.001) |
| **3** | 14 | 35.58 | 38.52 | -13,923.15 | 27880.40 | 27880.30 | **28012.88** | 27898.41 | **27995.88** | **27941.86** | 27920.24 | 447.246 (p <0.001) |
| **4** | 8 | 7.16 | 7.13 | -13,908.95 | **27864.08** | **27863.90** | 28043.28 | **27888.11** | 28020.26 | 27947.19 | **27917.94** | 27.293 (p <0.001) |
| **5** | 2 | 4.77 | 4.79 | -13,907.74 | 27873.77 | 27873.49 | 28099.66 | 27903.81 | 28070.66 | 27978.51 | 27941.62 | 0.255 (p =1.00) |

N class: number of classes, Df: Degrees of freedom, Gsq: Likelihood ratio / deviance statistic, χ2 LRT: likelihood ratio chi square goodness-of-fit; -LL: log likelihood, HT, Hurvich and Tsai Criterion, AIC: Akaike information criterion, CAIC: Consistent Akaike information criterion, AICc: Corrected Akaike Information Criterion, BIC: Bayesian information criterion, ssBIC: sample-size adjusted Bayesian information criterion, HQ, Hannan and Quinn Criteria, LMR-LRT: Lo-Mendell-Rubin ad-hoc adjusted likelihood ratio rest

**Table S2.**

*Item-response probabilities (ρ-estimate) and latent profile characterization of Latent profile analysis of smoking, binge drinking, cannabis consumption, and physical activity in 7,203 students.*

| **Item response probabilities** |  |  | |  |  |  |  |  |  |
| --- | --- | --- | --- | --- | --- | --- | --- | --- | --- |
| **Variable** | **Categories** | **ρ-estimate** | | **1** | **2** | **3** | **4** |  |  |
| **Smoking (avg. cigarettes / day)** | (almost) none or less than once a week | | | **0.9893** | 0.1885 | 0.2746 | **1.0000** |  |  |
| **Binge drinking (freq.)** | (almost) none or less than once a week | | | **0.8524** | **0.7287** | **0.5393** | **0.9013** |  |  |
| **Cannabis consumption (freq.)** | (almost) none or less than once a week | | | **0.9899** | **0.8411** | **0.6822** | **0.9921** |  |  |
| **Vigorous physical activity** | Once a week or daily | | | **0.9018** | 0.2428 | **0.8113** | 0.0042 |  |  |
| **Moderate physical activity** | Once a week or daily | | | **0.9410** | **0.6104** | **0.9528** | **0.7016** |  |  |
|  |  |  | |  |  |  |  |  |  |
| **Smoking (avg. cigarettes / day)** | Once a week or daily | | | 0.0107 | **0.8115** | **0.7254** | 0.0000 |  |  |
| **Binge drinking (freq.)** | Once a week or daily | | | 0.1476 | 0.2713 | 0.4607 | 0.0987 |  |  |
| **Cannabis consumption (freq.)** | Once a week or daily | | | 0.0101 | 0.1589 | 0.3178 | 0.0079 |  |  |
| **Vigorous physical activity** | (almost) none or less than once a week | | | 0.0982 | **0.7572** | 0.1887 | **0.9958** |  |  |
| **Moderate physical activity** | (almost) none or less than once a week | | | 0.0590 | 0.3896 | 0.0472 | 0.2984 |  |  |
|  |  |  |  | | | | | | |
| **Profile** | **Proportion** |  | **Description** | | | | | | |
| **1** | **53.0%** |  | **Health-protective behavior** | | | | | | |
| **2** | **4.2%** |  | **Moderate PA and smoking** | | | | | | |
| **3** | **14.2%** |  | **Licit and illicit substance use** | | | | | | |
| **4** | **28.6%** |  | **Moderate PA and no / low drug use** | | | | | | |
|  |  |  |  | | | | | | |
